# A Case of Severe Legionella Pneumonia in an Immunocompromised Traveler

**DOI:** 10.7759/cureus.82626

**Published:** 2025-04-20

**Authors:** Meaghan Bethea, Abdirahman Ahmed, Wayel Katrib

**Affiliations:** 1 Internal Medicine, Ross University School of Medicine, Pontiac, USA; 2 Internal Medicine, Trinity Health Oakland, Pontiac, USA

**Keywords:** early sepsis, immunocompromised patient, interstitial pneumonia, legionnaires, legionnaires' disease, waterborne disease, water exposure, water microbiology, water quality

## Abstract

Legionnaires’ disease is a severe pneumonia caused by *Legionella* species, typically acquired through inhalation of contaminated aerosols. Travel-associated cases pose a significant public health concern, particularly in resort settings where complex water systems may harbor the bacteria. We present the case of an immunocompromised 72-year-old woman who developed Legionnaires’ disease following a stay at a resort in Jamaica. Environmental investigations confirmed *Legionella* contamination in the resort’s water supply, highlighting the need for stringent water management practices in recreational facilities. This case underscores the importance of considering Legionnaires’ disease in patients with pneumonia and recent travel to high-risk environments. Early recognition and prompt treatment are crucial in reducing morbidity and mortality. Additionally, routine water testing and maintenance of resort water systems are essential preventive measures. Clinicians play a key role in identifying and reporting cases to facilitate timely public health interventions and outbreak prevention.

## Introduction

Legionnaires’ disease is a severe, potentially life-threatening form of pneumonia caused by *Legionella pneumophila* (*L. pneumophila*), an aerobic gram-negative bacillus commonly transmitted through inhalation of aerosolized water droplets from contaminated sources. This opportunistic pathogen thrives in warm, aquatic environments-particularly artificial water systems such as hot tubs, cooling towers, decorative fountains, and plumbing infrastructure-where inadequate maintenance promotes bacterial growth and dissemination [1,2]. Outbreaks are frequently linked to man-made water systems in public and recreational settings, making travel-associated cases a significant public health concern [3].

Although its overall incidence is relatively low compared to other pneumonias, *Legionella* is responsible for a notable proportion of severe community-acquired and travel-associated pneumonia worldwide [2]. In the United States alone, more than 10,000 cases are reported annually, with a case fatality rate of approximately 10% among hospitalized patients-higher in those with immunosuppression or delays in diagnosis and treatment [1]. The disease primarily affects older adults and individuals with underlying chronic illnesses or immunocompromising conditions.

Clinical manifestations typically include high fever, cough, dyspnea, and systemic symptoms such as myalgia, gastrointestinal disturbances, and altered mental status [1]. Because symptoms overlap with other respiratory infections, diagnosis often requires a high index of suspicion and confirmatory laboratory testing, such as urinary antigen assays or culture of respiratory specimens [1]. Early identification and prompt initiation of appropriate antibiotics, such as fluoroquinolones or macrolides, are crucial in improving patient outcomes [3].

This case report describes a patient who developed Legionnaires’ disease following a stay at a resort in Jamaica, emphasizing the importance of considering *Legionella* infection in travelers presenting with pneumonia. Furthermore, it highlights the public health implications of environmental exposure to contaminated water systems and the need for rigorous prevention and mitigation strategies in international hospitality settings.

## Case presentation

A 72-year-old woman presented to the emergency department with acute onset of delirium and high-grade fever. She reported flu-like symptoms, including cough, congestion, and generalized myalgia, that began four days earlier while staying at a resort in Jamaica. Of note, the patient reported participating in water activities while in Jamaica, including swimming in the community pool and hot tubs. Additionally, the patient sustained a cut on her foot from coral during her trip. She also noted multiple episodes of watery diarrhea but denied shortness of breath, chest pain, nausea, or vomiting. Approximately five weeks prior, her primary care physician had initiated methotrexate for her rheumatoid arthritis in addition to hydroxychloroquine. Additional past medical history included asthma, managed with montelukast and fluticasone furoate and vilanterol, hyperlipidemia, gastroesophageal reflux disease (GERD), and herpes simplex virus (HSV)-2 managed with valacyclovir. She had a 20 pack-year smoking history, quit several years ago, and was a social drinker.

On physical examination, the patient was febrile (104.7°F), tachypneic with a respiratory rate of 27 breaths per minute, and appeared acutely ill. Her oxygen saturation was 98% on room air. Lung auscultation revealed bilateral coarse crackles in the lower lung fields. Examination of her foot revealed a superficial laceration at the site of the coral scrape, without signs of localized infection such as erythema, swelling, or purulent discharge. Cardiovascular and abdominal examinations were unremarkable, but her altered mental status and systemic signs suggested a systemic inflammatory response. 

**Figure 1:**
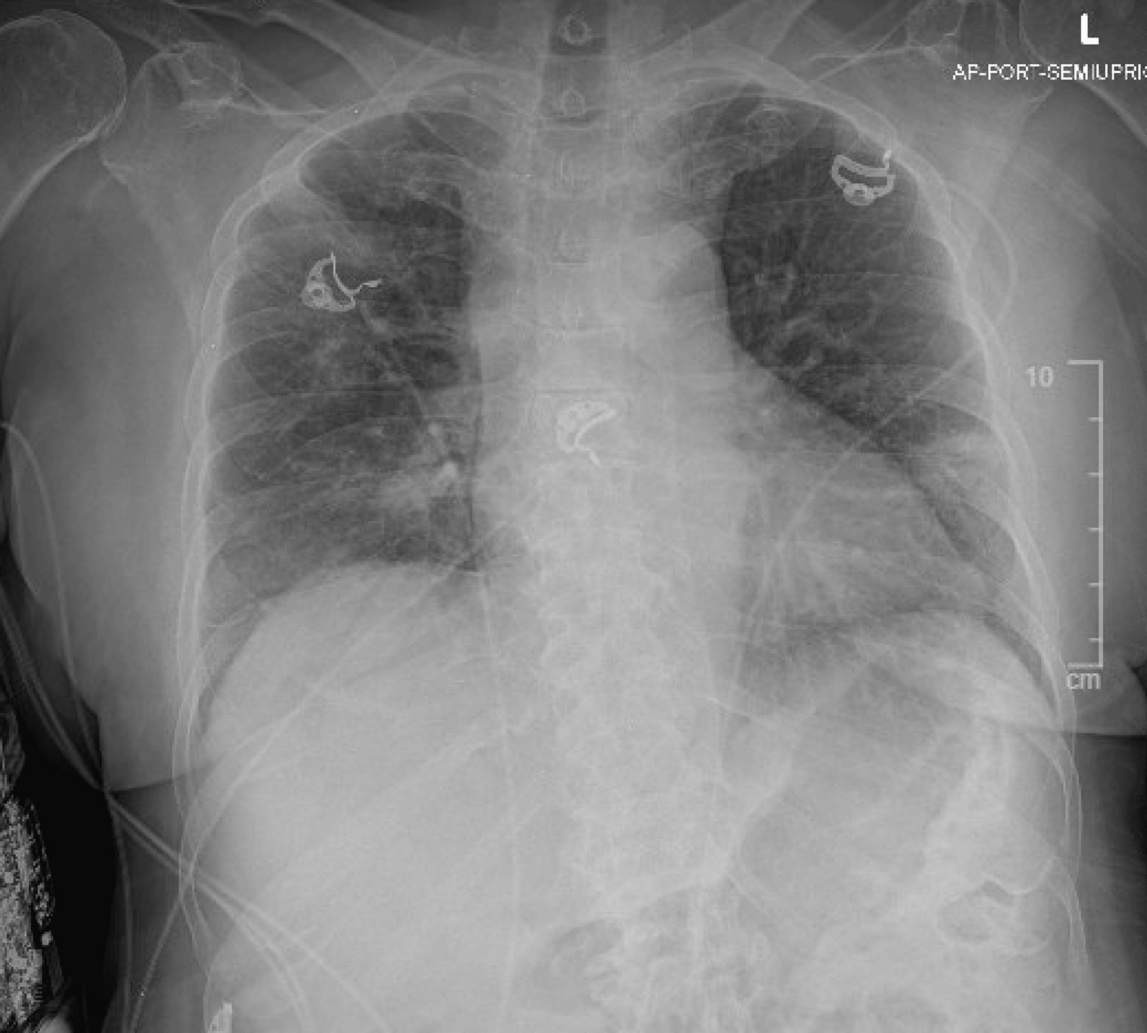
Chest radiograph demonstrating bilateral patchy airspace opacities, more pronounced in the left lower lobe, consistent with multifocal pneumonia. Cardiac silhouette and mediastinal contours are within normal limits. Medical devices and ECG leads are also visible.

Initial laboratory evaluation revealed leukocytosis (white blood cell count of 12,700/mm³) with absolute neutrophilia (11.25 K/mcl), mild hyponatremia (sodium 131 mmol/L), and an elevated procalcitonin level (1.61 ng/mL). Aspartate aminotransferase were mildly elevated. Ammonia and lactate levels were within normal limits. Venous blood gas analysis revealed mild hypoxemia with a normal pH. Chest x-ray imaging (Figure 1) demonstrated bilateral patchy infiltrates predominantly in the lower lung fields, raising suspicion for atypical pneumonia. Rapid viral screens for influenza, SARS-CoV-2, and respiratory syncytial virus (RSV) were negative. Initial blood cultures were pending at the time of admission.

The clinical picture of high fever, tachypnea, and leukocytosis with a left shift raised concerns for systemic inflammation secondary to an infectious etiology. Due to her extensive allergies to beta-lactams and cephalosporins, empiric intravenous levofloxacin was initiated to cover atypical pathogens. Additionally, IV fluids were administered to correct hyponatremia, and antipyretics were given for fever control. Given her history of recent coral exposure, the possibility of a secondary infection from marine pathogens such as *Vibrio *species or *Mycobacterium marinum* was considered. However, the lack of localized infection at the injury site made this less likely as a primary cause of her systemic illness. Considering her recent travel history and exposure to potential waterborne pathogens, further diagnostic testing was performed. A urinary antigen test for *L. pneumophila* was ordered and returned positive, confirming the diagnosis of Legionnaires’ disease. Blood cultures and additional testing for marine pathogens were monitored but ultimately yielded no growth. The patient was admitted to the hospital for further management, including continued antibiotic therapy and supportive care. Environmental investigations subsequently identified *Legionella* species in the water systems of the resort where she had stayed.

The patient’s condition improved significantly over the course of a five-day hospitalization. Her mental status returned to baseline on hospital day 2, she defervesced by hospital day 3, and by discharge, her respiratory status remained stable without the need for supplemental oxygen. She was discharged in stable condition with instructions for follow-up with her primary care physician and rheumatologist for continued monitoring and management of her underlying medical conditions.

Given the confirmed diagnosis of Legionnaires’ disease and the patient’s recent exposure to recreational water systems while staying at a resort in Jamaica, hospital staff contacted the resort to notify them of the potential public health concern. The notification included clinical details and a request for follow-up regarding any additional guest illnesses and environmental safety measures. Resort management acknowledged the report and initiated an internal investigation with the assistance of local environmental health authorities and an independent water safety consultant.

Environmental testing of the resort’s water systems was subsequently performed, including sampling from high-risk locations such as hot tubs, pools, guest room showers, air-conditioning units, and plumbing systems. Water samples were analyzed using culture methods and polymerase chain reaction (PCR) assays for *L. pneumophila*, which confirmed contamination with *L. pneumophila* serogroup 1 in both the hot tubs and sections of the guestroom water supply. These findings were consistent with the patient's clinical test results and supported the hypothesis that the resort's water system was the likely source of exposure.

In response, the resort implemented a multi-step remediation protocol, which included hyperchlorination of the plumbing systems, increasing water heater temperatures, flushing all fixtures, and temporary closure of the contaminated hot tubs. A commercial water treatment company was contracted to oversee remediation efforts and to install a long-term secondary disinfection system. Staff at the resort received additional training on *Legionella* prevention, water safety management, and risk communication. The resort also reviewed recent guest rosters and sent notifications to individuals who had stayed at the property during the potential exposure window. No other confirmed cases were reported at the time of this writing.

## Discussion

Legionnaires’ disease is a severe and potentially life-threatening pneumonia caused by *L. pneumophila*, typically acquired through inhalation of aerosolized bacteria from contaminated water systems [4]. Clinicians should maintain a high index of suspicion for *Legionella* infection in patients presenting with severe pneumonia and systemic inflammatory response syndrome, especially when there is a recent history of travel or exposure to communal water sources. Travel-associated cases are well-documented and frequently linked to hotels and resorts, where lapses in water system maintenance-such as inadequate disinfection, improper temperature regulation, and biofilm accumulation-create ideal conditions for bacterial proliferation [5].

In this case, the patient presented with altered mental status, high-grade fever, tachypnea, and bilateral pulmonary infiltrates, findings consistent with severe pneumonia and early systemic inflammatory response. Her recent participation in water activities at a resort in Jamaica raised clinical suspicion for waterborne pathogens, particularly *L. pneumophila*. The diagnosis was confirmed by a positive urinary antigen test, which is both rapid and highly sensitive for *L. pneumophila *serogroup 1, the most common cause of Legionnaires’ disease [6].

An additional complicating factor was a coral scrape sustained during her trip, raising concern for secondary marine infection. Coral injuries are known risk factors for infections by marine pathogens such as *Vibrio* species, *Mycobacterium marinum*, and *Erysipelothrix rhusiopathiae* [7]. Although these infections are typically localized, they can result in systemic illness in immunosuppressed individuals, such as this patient, who was receiving methotrexate and hydroxychloroquine for rheumatoid arthritis. However, the absence of localized signs of infection at the injury site and the predominantly pulmonary clinical picture made this a less likely etiology. Negative blood cultures for marine organisms further supported this conclusion. Nonetheless, this case underscores the need to consider polymicrobial or concurrent infections in immunocompromised hosts [8].

Treatment of Legionnaires’ disease requires prompt initiation of effective antibiotics that achieve high intracellular concentrations, such as fluoroquinolones or macrolides [9]. Intravenous levofloxacin was selected in this case due to the patient’s documented allergies to beta-lactams and cephalosporins and its proven efficacy against *Legionella*. The patient responded well to therapy, with resolution of fever, improvement in mental status, and stabilization of respiratory function over the course of her hospitalization.

Despite often being treatable, Legionnaires’ disease carries significant morbidity and mortality, particularly in older adults and immunocompromised individuals. While most patients recover with appropriate antibiotic therapy, complications such as respiratory failure, septic shock, and multi-organ dysfunction may occur [10]. Long-term sequelae-including persistent fatigue, dyspnea, and cognitive impairment-have been reported in up to 40% of survivors following hospitalization [11]. Though relapse is rare, it can occur, especially in patients with structural lung disease or ongoing environmental exposure [12]. These risks emphasize the importance of administering an adequate course of antibiotics, typically seven to 14 days depending on severity and comorbidities, and ensuring close outpatient follow-up to monitor symptom resolution and pulmonary function [9,12].

From a public health standpoint, this case highlights the need for more stringent regulations, routine surveillance, and standardized protocols for water system maintenance in recreational and hospitality settings. Travel-associated Legionnaires’ disease remains a global concern, with outbreaks frequently linked to resorts and hotels due to suboptimal oversight. Environmental testing confirmed the presence of *Legionella* in the resort’s water system, highlighting a preventable risk factor for disease transmission.

To mitigate future risk, resorts and similar facilities must adopt comprehensive water safety plans that include regular disinfection protocols, strict temperature regulation-since *Legionella* thrives between 25°C and 45°C-and scheduled microbial testing [2]. Upon notification of this case, the resort in Jamaica was informed of the environmental findings, prompting an internal review of water maintenance practices. While this investigation did not confirm additional cases, increased vigilance and remediation efforts were implemented to reduce the risk of future outbreaks.

Broader strategies should include collaboration between international travel authorities and public health agencies to ensure swift case notification, prompt environmental testing, and transparent remediation. Education of resort operators on infection prevention and traveler awareness campaigns may further contribute to decreasing the burden of travel-associated Legionnaires’ disease. Ultimately, this case demonstrates how a single clinical encounter can prompt wider public health action, turning individual illness into an opportunity for systemic improvement.

## Conclusions

This case of travel-associated Legionnaires’ disease in an immunocompromised older adult highlights the critical role of thorough clinical history-including environmental exposures and recent travel-in guiding timely diagnosis and management. Prompt identification using a urinary antigen test and appropriate antibiotic therapy led to clinical improvement and recovery. Additionally, attention to other exposures, such as coral scrapes, ensures that less common but potentially significant pathogens are considered, particularly in immunocompromised patients. From a public health standpoint, the identification of *Legionella *in the resort’s water systems reinforces the need for stricter international standards and proactive monitoring of recreational water environments. Facilities catering to travelers must prioritize routine water quality control and rapid response protocols to minimize preventable exposure risks. Clinicians should maintain a high index of suspicion for *Legionella* in patients presenting with pneumonia and relevant exposure histories. Ultimately, this case exemplifies how early clinical recognition and collaborative public health measures can improve individual outcomes and reduce the broader burden of travel-associated infectious diseases.
